# Leaf Age, Canopy Position, and Habitat Affect the Carbon Isotope Discrimination and Water-Use Efficiency in Three C_3_ Leguminous *Prosopis* Species from a Hyper-Arid Climate

**DOI:** 10.3390/plants8100402

**Published:** 2019-10-09

**Authors:** M. Iftikhar Hussain, Ali El-Keblawy, François Mitterand Tsombou

**Affiliations:** 1 Research Institute of Science and Engineering (RISE), University of Sharjah, Sharjah 27272, UAE; akeblawy@sharjah.ac.ae; 2 Department of Applied Biology, Faculty of Science, University of Sharjah, Sharjah 27272, UAE; fmitternanatsombou@sharjah.ac.ae; 3 Departmento de Biología Vegetal, Universidad de Málaga, 29016 Málaga, Spain

**Keywords:** carbon isotope composition, leaf C, leaf N, intrinsic water-use efficiency, canopy position, leaf age

## Abstract

The present study involved measurements of the stable carbon isotope composition (δ^13^C) and intrinsic water-use efficiency (iWUE) of three C_3_ leguminous *Prosopis* spp. (*P. juliflora*, *P. cineraria*, and *P. pallida*) foliage at different canopy positions (east and west) from saline (SLH) and non-saline habitats (NSH). Integrated measurements of the stable carbon isotope composition (δ^13^C) of plant tissue were broadly used to study iWUE, taking into consideration the effect of leaf age and canopy position on C isotope discrimination. Mature foliage of *P. pallida* from an SLH with a west canopy position had significantly higher δ^13^C (less negative) than that from NSH. On the west side, Δ^13^C values ranged from 17.8‰ (*P. pallida*) to 22.31‰ (*P. juliflora*) for a west canopy position, while they varied from 18.05‰ (*P. pallida*) to 22.4‰ (*P. cineraria*) on the east canopy side. Because the patterns are similar for the three *Prosopis* species, the difference in carbon isotope discrimination (Δ^13^C) between the canopy position (west and east) is relatively consistent among species and sites, ranging between 17.8 ± 4.43‰ for the young foliage in the west and 18.05 ± 4.35‰ for the east canopy position. The iWUE of *P. pallida* was twice that of *P. cineraria*. The iWUE of *P. juliflora* was higher from NSH than SLH. Mature leaves possessed a higher iWUE than the young leaves. We concluded that exotic *P. juliflora* and *P. pallida* have higher iWUE values than the native *P. cineraria*, which might be due to the rapid below-ground development of plant roots in the Arabian deserts of the United Arab Emirates (UAE). This could enable the alien species access to deeper humid soil layers or water resources.

## 1. Introduction

The element carbon possesses three naturally occurring isotopes: ^12^C, ^13^C, and ^14^C. Usually, the focus is on the first two carbon forms, which are stable isotopes (^12^C and ^13^C), because they are differently fractionated by photosynthetic pathways. A difference in isotope assimilation into the plant structure is mainly attributed to a difference in the diffusion rates of ^12^CO_2_ and ^13^CO_2_ during the photosynthetic assimilation of CO_2_, in conjunction with carboxylation enzyme preference for one isotopic form of carbon dioxide over another [1]. Photosynthetic assimilations of CO_2_ and carboxylation enzyme preference are dependent upon the photosynthetic mechanisms that each plant uses—either C_3_ or C_4_ in this case. In regards to C_3_ plants, Rubisco enzymes demonstrate a large kinetic isotopic effect that favors the assimilation of ^12^CO_2_ over ^13^CO_2_. Stable isotope analysis has provided crucial insights for understanding connections between the soil, vegetation, and atmosphere. The study of the stable isotopes of carbon, nitrogen, and oxygen composition provides further understanding of the biogeochemical cycle of plant ecosystems. Previously, several researchers, including Farquhar et al. [1,2], Seibt et al. [3], and Cernusak et al. [4], have proposed a theory that explains the δ^13^C composition of plant photosynthetic CO_2_ very well through gas exchange measurements or indirectly, from the organic matter of the plants. Plant photosynthesis discriminates against the stable ^13^C isotope when atmospheric CO_2_ passes through stomata and during CO_2_ carboxylation in RuBisCO [5]. The variations in stable isotopes of C and N provide potentially valuable information regarding the phenotypic plasticity, because stable isotope ratios are reliable indicators of spatially and temporally dynamic changes in the environment. The plant water-use efficiency can be measured at different levels, such as the leaf, whole plant, and ecosystem levels [1]. Water-use efficiency is a measure of the amount of water used by plants per unit of plant material produced. At the leaf level, it is more precisely referred to as “intrinsic water-use efficiency”, “instantaneous water-use efficiency”, or “transpiration efficiency” (TE) [1,2], and represents the moles of CO_2_ utilized during photosynthesis (*A*) divided by the moles of water lost through transpiration (*T*) per unit time and unit leaf area. At the whole-plant level, water-use efficiency is referred to as the units of dry matter production divided by the units of water lost [1,4,5]. The water-use efficiency (WUE) demonstrates photosynthetic C accumulation and the loss of transpirational water from the plant. The WUE is an important surrogate for evaluating the biological fitness, yield, plant productivity, and physiological plasticity during a plant–ecosystem exchange process [2,3]. Meanwhile, several environmental factors (biotic and abiotic stress) influence the plant WUE and include the genetic variability, growth stage, climatic conditions, and ecosystem types. These factors ultimately impact the stomatal conductance and photosynthetic efficiency and hence lead to changes in the WUE [1,2].

The genus *Prosopis*, with 44 species of medium-sized trees and shrubs, belongs to the family *Fabaceae*, and subfamily *Mimosoideae*. Hyper-arid climates have significant amounts of barren sand dunes and established halophytic species; the study of different *Prosopis* species offers special opportunities to clarify variations in plant and ecosystem physiology because of the wide range of environmental conditions in different seasons [6]. Some species of *Prosopis* are important for local communities as they provide numerous goods and services, such as a honey source, fuel, and wood. Despite the fact that most species of *Prosopis* are beneficial, other species generate benefits and costs. Because of the great environmental and economic benefits, the *Prosopis* genus has attracted increasing attention in ecological research [6]. In this study, we selected three *Prosopis* species (*Prosopis juliflora*, *Prosopis cineraria*, and *Prosopis pallida*), which are dominant tree species in hyper-arid desert regions [7], including in the United Arab Emirates (UAE). Pennington et al. [8] previously analyzed the δ^13^C and iWUE from *Prosopis glandulosa* Torr. from adult trees distributed along a wide precipitation gradient that included mesic and semi-arid grassland, and Chihuahuan desert ecosystems. They reported that *P. glandulosa* showed more negative δ^13^C (indicative of a lower WUE) from the Chihuahuan desert compared to plants from other habitats. Moreover, plants from drier, western extremes were better adapted than those from mesic areas. Sun et al. [9] (2009) studied the diurnal and seasonal patterns of δ^13^C in *Prosopis velutina* from flood plains and upland savanna ecosystems in southwestern USA. The δ^13^C values increased during the daytime and then gradually declined during nighttime periods to minimum values at pre-dawn. The magnitude of the diurnal shift in δ^13^C was due to the season and habitat, soil water, and leaf level vapor pressure deficits. In this study, we study whether the foliar δ^13^C and water-use efficiency of these three *Prosopis* species showed positive relationships with leaf age and canopy position from saline and non-saline habitats. Utilizing these three C_3_ *Prosopis* spp. (*P. juliflora*, *P. cineraria*, and *P. pallida*) as a model leguminous system in an arid desert environment, this study aimed to investigate the occurrence and extent of carbon isotope discrimination between young and mature leaf carbon pools on an organic matter basis and evaluate the magnitude of changes for predictions of WUE. As two of the *Prosopis* spp. are invasive exotic (*P. juliflora* and *P. pallida*) and the third is native (*P. cineraria*), this study presents the chance to compare the water-use efficiency of exotic and invasive *Prosopis* species.

## 2. Material and Methods 

### 2.1. Study Sites

The study was conducted at five locations in the northeastern UAE (Kalba, University of Sharjah (UoS), Ajman, Qarina, and Umm al-Quwain (UmQu)). These sites contain native and introduced exotic invasive *Prosopis* spp. We studied two exotic *Prosopis* species (*P. juliflora* and *P. pallida*) and one native species (*P. cineraria*). The species were sampled from 60 wild populations (20 for *P. juliflora* and *P. pallida*) across saline habitats (SLH) and 20 for *P. cineraria*, from the non-saline habitats (NSH). In Sharjah, the mean annual temperature is approximately 26.99℃, with a maximum temperature of 34.7℃. Geographical information and altitude were recorded using a global positioning system. Meteorological data of each sampling site were obtained from the online portal of the International Water Management Institute (IWMI) (http://wcatlas.iwmi.org/Default.asp). All of the climatic data are reported in Table S1. No specific permissions were required to conduct the field studies described at each particular site and the study was conducted in accordance with the guidelines set by the University of Sharjah, Sharjah, United Arab Emirates. Furthermore, none of the sites were privately owned or protected area field studies and did not involve endangered or protected species. At each sampling site, we collected the leaves from each *Prosopis* spp. from the east and the west canopy side of each tree. Samples from individual trees 6 m apart from each other were collected and pooled into one sample. A minimum of six replicates from each population was collected and all samples were collected from robust mature trees (10–15 years old). 

### 2.2. Leaf C and N Content

The leaf samples were dried at 65℃ until reaching a constant weight and were then ground to a fine powder. An aliquot of 1 mg dry powder was analyzed for the C (%) and N content (%) with an Elemental Analyzer (Flash EA-1112, Swerte Germany).

### 2.3. Plant Sampling and Data Collection

The leaf samples from all three *Prosopis* spp. were placed in paper bags and dried at 70℃ for 72 h in a forced-air oven and ground in Ball Mills. δ^13^C values were determined from the dry and ground samples (1700–2100 µg; filled in tin capsules), combusted at 1600–1800℃ in the presence of oxygen, and converted to CO_2_ using an Isotopic Ratio Mass Spectrometer (Finnegan: Thermo Fisher Scientific, model MAT-253, Swerte Germany).

Stable C isotope ratios (δ^13^C) are demonstrated as per mil (‰), which is a measure of the ^13^C/^12^C ratio in a plant sample relative to same ratio in limestone (Pee Dee belemnite):

δ^13^C(‰) = [(R_sample_/R_standard_) —1)] x 1000 (1)

where R_sample_ is the isotopic ratio (^13^C/^12^C) and R_standard_ is the Vienna PeeDee Belemnite (VPDB) standard for the carbon. The analytical precision of the laboratory standards was better than 0.3‰ for the ^13^C. The standard carbon R was equal to 1.1237 x10^−2^. Pure CO_2_ (δ^13^C = −28.2 ± 0.1‰) gas calibrated against the standard CO_2_ (−10.38‰) served as the reference gas for δ^13^C. The accuracy and reproducibility of the measurements of δ^13^C were checked with an internal reference material (NBS 18 and IAEA-C6 for C) and acetanilide for the C percentage, respectively. Carbon isotope discrimination (Δ^13^C), the intercellular CO_2_ concentration from inside to ambient air (*C*i*/C*a), and the intrinsic water-use efficiency (iWUE) were derived from the δ^13^C basic data, as reported previously [2,10,11,12]. Carbon isotope measurement was conducted at the Department of Servicios Centrales de Apoyo a la Investigación (SCAI), at Universidad de Málaga, Spain.

### 2.4. Data Analysis

The data was statistically analyzed using SPSS (SPSS Inc., Chicago, IL, USA, version 21.0). For all the data, a General Linear Model (GLM) with three-way ANOVA was produced, with four factors (plant species, canopy positions, habitat types, and leaf age) as functional traits and variables. Carbon isotope composition data was analyzed and the results are presented as the mean ± standard error (SE). Significant differences in the mean values were determined using Duncan’s multiple range test (p < 0.05). Graphical representation was generated by MS Excel.

## 3. Results

**Figure 1:**
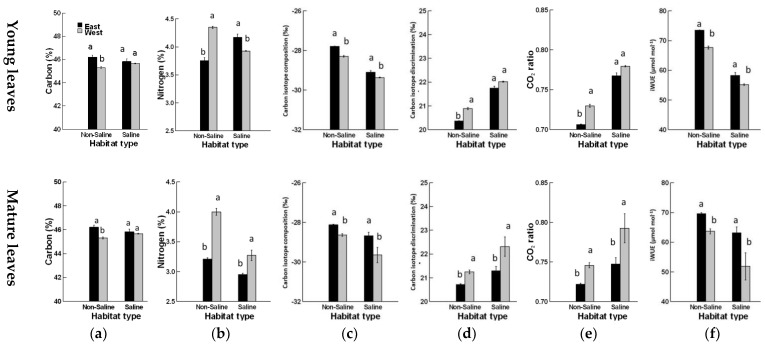
Effects of habitat type (saline vs. non-saline), canopy position (east and west) and leaf age (young and mature) on (**a**) carbon (%), (**b**) nitrogen (%), (c) carbon isotope composition (‰), (d) carbon isotope discrimination (‰), (**e**) *C*i/*C*a ratio, and (**f**) intrinsic water-use efficiency (iWUE) of *Prosopis juliflora.*

The effect of habitat type, age, interactions between the habitat type and both position and age, and the interaction between the canopy and age on the carbon contents were significant (*p <* 0.01). For nitrogen (%), all three factors and their interactions had significant effects (*p <* 0.05, Table 1). In NSH, the carbon (C%) in both young and mature leaves of *P. juliflora* was higher for the east than the west canopy position, while it remained unaffected in SLH (Figure 1a). There was a significant reduction in N% in young foliage on the east side compared to the west canopy position in NSH. The opposite was true in SLH, where the N% significantly increased on the east side compared to the west canopy position. In mature leaves, N% was significantly increased on the west side compared to the east canopy position in both NSH and SLH, and this difference was more than 45%.

There were significant effects for both canopy position and habitat, but not for the age or any of the interactions between leaf carbon isotope attributes and iWUE (Table 1). The highest values (least negative) of leaf carbon isotope composition (δ^13^C) were observed in *P. juliflora* from both young and mature leaves in NSH for the east canopy position (Figure 1b). A similar pattern of variation was observed in δ^13^C in *P. juliflora* from both young and mature leaves in SLH. Carbon isotope discrimination (Δ^13^C) values were significantly higher for the west canopy position in both young and mature foliage in both habitats. The effects of different habitats on *C*i/*C*a varied among young and mature leaves and among the individual *Prosopis* spp. The *C*i/*C*a decreased significantly on the east side of *P. juliflora*, with higher values obtained on the west side from both young and mature foliage. The iWUE in the young foliage was greater in *P. juliflora* for the east canopy position from NSH than SLH. Moreover, young leaves possessed higher iWUE than mature leaves (Figure 1f).

The effects of habitat, but not canopy position, and the interaction between habitat and canopy position, on all studied attributes of *P. pallida* were significant (Table 2). Carbon (C%) and nitrogen (N%) contents in mature leaves of *P. pallida* were higher for the east than the west canopy position, while the difference within the canopy position was more prominent in N%, which was three-fold higher in the east than west. An opposite trend was observed in SLH, where C% significantly increased for the west compared to east canopy position. In SLH, N% was eight-fold higher at the west canopy position than at the east.

**Figure 2:**
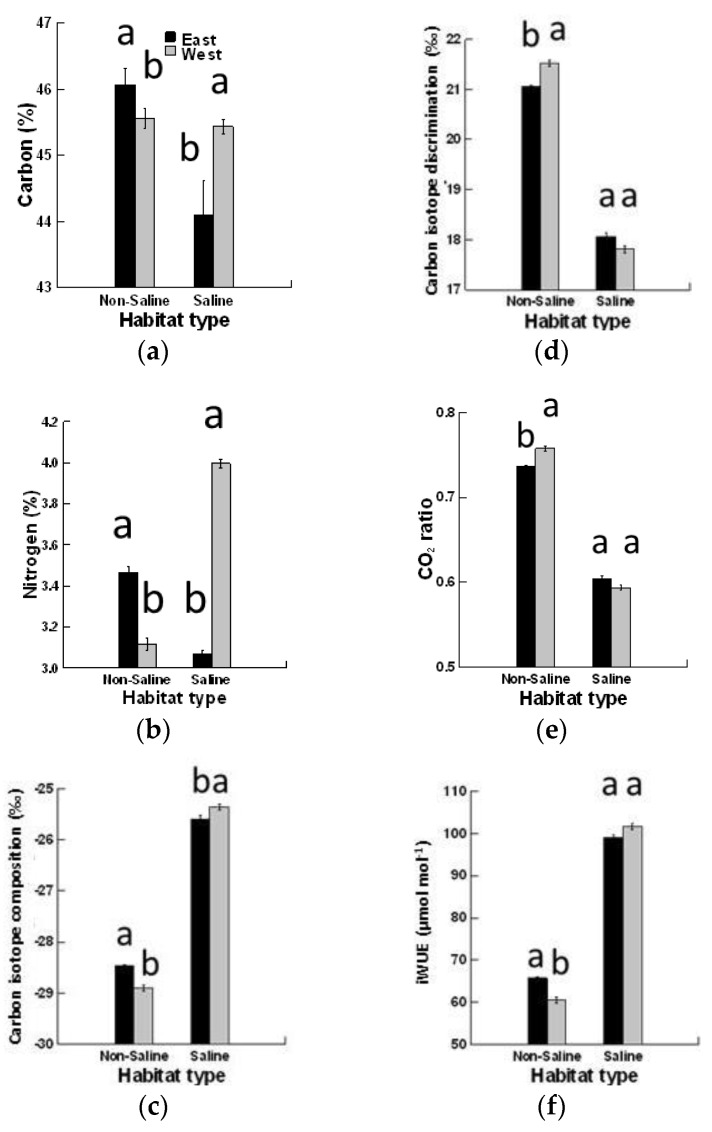
Effects of canopy position (east and west) and habitat (saline vs non-saline) on (**a**) carbon (%), (**b**) nitrogen (%), (**c**) carbon isotope composition (‰), (**d**) carbon isotope discrimination (‰), (**e**) *C*i/*C*a ratio, and (**f**) intrinsic water-use efficiency of mature leaves of *Prosopis pallida.*

The highest values (least negative) of leaf δ^13^C were observed in *P*. *pallida* in mature leaves from the saline habitat on both tree sides compared to the non-saline habitat (Figure 2). *P. pallida* demonstrated the lowest (most negative) values of δ^13^C in mature leaves that differ significantly from the west side when compared to the east canopy position. In NSH, Δ^13^C values in *P. pallida* ranged from 21.06‰ (east canopy position) to 21.53‰ (west canopy position), while they varied from 17.8‰ (west canopy position) to 18.05‰ (east canopy position) in SLH. The Δ^13^C was higher in the non-saline (22.23‰) compared to saline habitat (17.8‰). The *C*i/*C*a decreased significantly on the east side compared to the west side under the NSH. Under a saline environment, mature foliage possessed higher *C*i/*C*a values for the east canopy position compared to the west side. The *C*i/*C*a values were greater in NSH than SLH. Under a saline habitat, a highly significant increase in iWUE in *P. pallida* mature foliage was obtained on the west (101.67 μmol CO_2_ mol H_2_O^−1^) and east sides (98.93 μmol·CO_2_ mol H_2_O^−1^). The lowest iWUE values were obtained from mature foliage from NSH (Figure 2).

**Figure 3:**
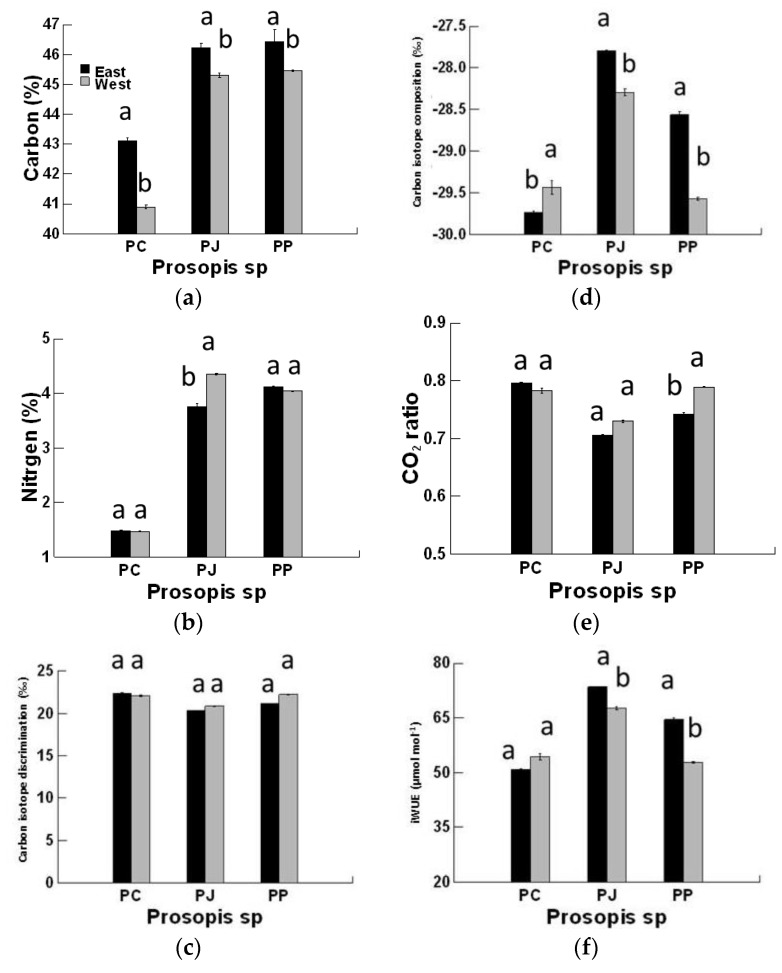
Effects of *Prosopis* species (*Prosopis juliflora*, *Prosopis pallida*, and *Prosopis cineraria*) canopy position (east and west) on (**a**) carbon (%), (**b**) nitrogen (%), (**c**) carbon isotope composition (‰), (**d**) carbon isotope discrimination (‰), (**e**) *C*i/*C*a ratio, and (**f**) intrinsic water-use efficiency of young leaves of three species in the non-saline habitat.

The *Prosopis* species type and canopy position and their interactions had significant effects on the studied attributes (Table 3). In the young foliage, the carbon (C%) content was significantly higher at the east canopy position compared to the west in the three species. The N% had significantly lower values in young foliage of *P. juliflora* at the east compared to the west canopy position. In both the exotic *P. juliflora* and *P. pallida*, the N content (%) was three-fold higher than in the native *P. cineraria* (Figure 3).

There was significant variation in the carbon isotope composition (δ^13^C) in young leaves within the three studied *Prosopis* spp. The highest values (least negative) of leaf δ^13^C were observed in *P. juliflora* and *P. pallida* at the east compared to west canopy position (Figure 3). Compared to the two exotic species, the *P. cineraria* showed the lowest value of δ^13^C in young leaves; this was significantly greater at the west side than at the east side of the canopy. From the east to west side, individual carbon isotope discrimination (Δ^13^C) values varied from 20.35‰ to 22.23‰ (*P. juliflora*) and 21.17‰ to 22.23‰ (*P. pallida*), but there was no variation in the two canopy positions of the *P. cineraria* (22.4‰–22.08‰) (Figure 3).

The *C*i/*C*a values remain un-affected on either side of the canopy in *P. juliflora* (Figure 3). The *P. pallida* young foliage possesses higher *C*i/*C*a values on the west side compared to the east side, but an opposite trend was observed for *C*i/*C*a in young leaves of *P. cineraria*. This indicates that *P. pallida*’s west canopy position might not suffer from excess light and heat stress, and higher N fixation/availability might stimulate CO_2_ carboxylation in RuBisCO. On the west side, individual *C*i/*C*a values ranged from 0.59 (*P. pallida*) to 0.79 (*P. juliflora*), while they varied from 0.6 (*P. pallida*) to 0.79 (*P. cineraria*) on the east canopy side. 

The iWUE values of 52.75–64.49 μmol·mol^−1^ were obtained from the east to west in the young foliage of *P. pallida.* In *P*. *cineraria*, the iWUE in young foliage on the west canopy side was 54.32 μmol·mol^−1^ and 50.87 μmol·mol^−1^ on the east canopy side. The iWUE in *P. juliflora* young foliage on the west side was 67.65 and 73.47 μmol·mol^−1^ on the east side. Meanwhile, our results clearly demonstrate that under the NSH, the iWUE in *P. juliflora* was 30% higher than *P. cineraria* at the east canopy position (Figure 3).

## 4. Discussion

Carbon isotope ratios are considered effective tools for detecting environmental stresses in various crops, weeds, vegetables, and herbaceous plants [11,12,13]. Changes in the isotopic signatures of plant dry matter are very useful for studying the CO_2_ assimilation (photo-respiration during the day-time) and discrimination that can be detected during night-time respiration [1,2]. We studied three *Prosopis* spp. (*P. juliflora*, *P. cineraria*, and *P. pallida*) across saline and non-saline habitats from the hyper-arid regions of the United Arab Emirates. The native tree *P. cineraria* and the invasive trees *P. juliflora* and *P. pallida* showed contrasting physiological and isotopic responses due to their leaf age, canopy position, and interaction. The foliar δ^13^C values of the three *Prosopis* spp. fell within a range of δ^13^C that has been reported for other C_3_ leguminous species (0 + 2‰) [13]. We found a significant difference in foliar δ^13^C among the three species that could be due to the variation in foliar δ^13^C from the canopy positions (east to west). Meanwhile, leaf age and habitat (SLH and NSH) have a tight correlation for δ^13^C. We observed the highest δ^13^C values at the west canopy positions compared to the east in *P. pallida* from the saline habitat. It seems that, in saline habitats, plants might suffer from water limitation, less photosynthesis due to stomatal closure, and hence more carbon isotope discrimination. In the case of *P. juliflora*, we found the lowest δ^13^C values in mature leaves from the west canopy position of trees from the saline habitat. 

Stable carbon isotope (δ^13^C) measurement is an important biological and physiological tool that provides an integrated measurement which helps trace carbon fluxes at an ecosystem scale along an environmental gradient. On the west side, individual carbon isotope discrimination (Δ^13^C) values ranged from 17.8‰ (*P. pallida*) to 22.31‰ (*P. juliflora*), while they varied from 18.05‰ (*P. pallida*) to 22.4‰ (*P. cineraria*) on the east canopy side. Highly ^13^C-enriched values of leaf dark-respired CO_2_ compared with primary respiratory substrates was observed in a number of species and across a range of environmental conditions [14,15]. Changes in ^13^C enrichment occur over very short (minute) [16,17] and diurnal time scales [15,18,19]. We observed that Δ^13^C was higher in young foliage from NSH and it was discriminated at a rate of 4.43‰.

Carbon isotope discrimination was used to assess the intrinsic water-use efficiency and therefore served as a proxy for the determination of osmotic stress in plants [10,11,12]. In *P. pallida*, the *C*i/*C*a values were lower (0.59) in mature foliage from SLH than NSH (0.78) compared to young foliage. The *P. pallida* canopy position on the west side might not suffer from excess light and heat stress, and higher N fixation/availability might stimulate CO_2_ carboxylation in RuBisCO. A lower *C*i/*C*a ratio could result either from stomatal closure induced by water stress or from higher rates of photosynthetic capacity, or from a combination of both [20]. Meanwhile, iWUE in *P. pallida* was twice that in *P. cineraria*. Furthermore, the iWUE of *P. juliflora* was higher in NSH than SLH. Moreover, mature leaves possessed higher iWUE values than the young leaves. Plants from the contrasting ecosystems and genotypes of the same plants have different carbon isotope discrimination and water-use efficiencies because of genetic variation in the leaf gas exchange characteristics and because of variation in the environmental conditions among habitats [2,10,12,20,21,22]. Leaf carbon isotope composition (δ^13^C) has long been promoted as a proxy for an integrated measurement of transpiration efficiency (TE) in species [1,2,3,5,10]. Zolfaghar et al. [20] demonstrated that Δ^13^C can be used as a proxy for iWUE study in eucalyptus trees. Several authors have reported that iWUE is positively correlated with Δ^13^C [1,2,10]. C_3_ is a specialized mode of photosynthesis that exhibits CO_2_ uptake, facilitates an increased intrinsic water-use efficiency (iWUE), and enables three C_3_ leguminous *Prosopis* spp. to inhabit water-scare conditions such as hot-arid UAE deserts. The measurements of water-use efficiency and the ratio of carbon gain in net photosynthesis to water loss during transpiration, are critical to the survival, productivity, and biological fitness of plants, with implications for ecosystem energy exchange processes [10,14,17].

While acclimation of the leaves to arid environments, soil water deficit, and drought stress have been extensively studied [23,24], leaf structure, leaf age, and canopy position with respect to the light environment have received much less attention. We found significant differences between leaf age, canopy position, and *Prosopis* spp. for δ^13^C in the young and mature leaves at a species level. The differences in δ^13^C of leaf organic matter coincided with the canopy position and habitat and these differences appear sensitive to changes in the environmental conditions. Consequently, changes in leaf age were coupled with differences in species-specific and canopy position oriented-δ^13^C that was found to significantly influence predictions of iWUE based on isotopes. In the present study, we found that both invasive species *P. juliflora* and *P. pallida* have higher iWUE values than the native *P. cineraria* on both canopy sides. There might be rapid below-ground development of plant roots in *Prosopis pallida* in the Arabian deserts of the UAE that could enable the alien species access to deeper humid soil layers or water resources. This functional trait has been previously associated with a high invasive capacity in desert areas [25]. Another invasive *Prosopis* species, *P. glandulosa*, has been significantly established in East Africa in ephemeral riverbeds, saline lands, and abundant farms with deep soils and high water availability [26]. The successful establishment of alien invasive species in the microenvironments was possibly due to their early root establishment and easy access to humid soil layers [27]. In the arid lands of Arabia, *P. juliflora* dominates areas with a shallower water table [28]. These functional properties of exotic *Prosopis* species foster their adaptability and support their invasion across various agro-ecosystems, including wetlands, dry lands, and irrigated agricultural lands [29]. Careful examination of the *P. juliflora* invasion showed the presence of *P. juliflora* growing mixed with *P. pallida* in most of the invaded habitats of the UAE and this complex is highly aggressive and coppices so well that it crowds out native vegetation [30].

From the present study, we concluded that carbon isotope discrimination and intrinsic water-use efficiency are important ecological indicators that can be affected by the canopy position (west and east) among three C_3_ *Prosopis juliflora*, *Prosopis cineraria*, and *Prosopis pallida* from saline and non-saline habitats. The foliage from all three *Prosopis* spp. showed significant variation among carbon isotope discrimination and intrinsic water-use efficiency. Further studies are needed to collect material and associated metadata (tree position and height) from different age and size cohorts to investigate the possible intra-canopy effects on long-term trends in stable isotope ratios.

## Figures and Tables

**Table 1:** Results of two-way ANOVA (F-values) testing the effects of habitat type, canopy position, and leaf age on carbon (%), nitrogen (%), carbon isotope discrimination, and intrinsic water-use efficiency (iWUE) attributes of *Prosopis juliflora.*

Source of Variation	df	Carbon (%)	Nitrogen (%)	C Isotope Composition	C Isotope Discrimination	Ci/Ca Ratio	iWUE
Habitat (H)	1	93.13***	55.98***	80.37***	80.13***	79.35***	80.18***
Canopy Position (C)	1	0.839	122.08***	25.97***	25.89***	25.93***	25.92***
Age (A)	1	152.71***	438.32***	1.483	1.475	1.470	1.483
H*C	1	16.37**	97.85***	0.254	0.258	0.220	0.261
H*A	1	89.82***	54.78***	3.498	3.481	3.457	3.468
C*A	1	14.07**	32.90***	2.658	2.656	2.666	2.666
H*C*A	1	0.637	7.88*	2.505	2.498	2.562	2.509
Error	16						

* *p* < 0.05, ** *p* < 0.01, *** *p* < 0.001.

**Table 2:** Results of two-way ANOVA (F-values) testing the effects of habitat type (saline vs. non-saline) and canopy position (east and west) on carbon (%), nitrogen (%), carbon isotope discrimination, and intrinsic water-use efficiency (iWUE) of mature leaves of *Prosopis pallida.*

Source of Variation	df	Carbon (%)	Nitrogen (%)	C Isotope Composition	C Isotope Discrimination	Ci/Ca Ratio	iWUE
Habitat (H)	1	12.54**	90.88***	3097***	3105***	3025***	3105***
Canopy Position (C)	1	2.026	130.96***	3.155	3.233	3.422	3.233
H*C	1	9.672*	639.06***	34.65***	34.81***	33.58***	34.81***
Error	8						

* *p* < 0.05, ** *p* < 0.01, *** *p* < 0.001.

**Table 3:** Results of two-way ANOVA (F-values) testing the effects of *Prosopis* species (*P. juliflora*, *P. pallida*, and *P. cineraria*) and canopy position (east and west) on carbon (%), nitrogen (%), carbon isotope discrimination, and intrinsic water-use efficiency (iWUE) of young leaves of the three *Prosopis* species in the non-saline habitat.

Source of Variation	df	Carbon (%)	Nitrogen (%)	C Isotope Composition	C Isotope Discrimination	*C*i/*C*a Ratio	iWUE
Species (Sp)	2	286.73***	7737***	743.1***	742.12***	722.91***	742.12***
Canopy Position (C)	1	80.92***	74.1***	147.99***	147.64***	145.23***	147.64***
SP * C	2	7.948**	118.83***	130.26***	130.28***	125.79***	130.28***
Error	12						

* *p* < 0.05, ** *p* < 0.01, *** *p* < 0.001.

## Supplementary Materials

The following are available online at https://www.mdpi.com/2223-7747/8/10/402/s1, Table S1. Details of climatic data for each sampling locations across United Arab Emirates.

## Data accessibility

The data are provided in the same manuscript file.
